# Predicting Adverse ICU Outcomes from Admission-Time Frailty and Nocturnal Sleep Fragmentation Using Explainable AI

**DOI:** 10.3390/jcm15145538

**Published:** 2026-07-15

**Authors:** Bibars Amangeldy, Assiya Boltaboyeva, Zhanel Baigarayeva, Baglan Imanbek, Nurdaulet Tasmurzayev, Shugyla Kurmanbek, Sultan Tuleukhanov, Vyacheslav Lokshin

**Affiliations:** 1 AlfaCenter (Al-Farabi AI Center), Farabi University, Almaty 050040, Kazakhstan; 2 LLP “Kazakhstan R&D Solutions”, Almaty 050056, Kazakhstan; 3 Faculty of Biology and Biotechnology, Farabi University, Almaty 050040, Kazakhstan; 4 LLP “PERSONA International Clinical Center for Reproductology”, Almaty 050060, Kazakhstan

**Keywords:** frailty, sleep deprivation, intensive care units, machine learning, explainable artificial intelligence, electronic health records, critical illness, delirium, hospital mortality, respiration, artificial, risk assessment, treatment outcome, nursing care, databases, factual, prognosis

## Abstract

**Background**. Sleep fragmentation and frailty are interrelated yet underexplored determinants of adverse outcomes in critically ill patients, and no published model has combined objective nocturnal fragmentation metrics with administrative frailty indices to predict adverse outcomes at ICU admission. **Methods**. Using 31,139 first ICU admissions from the MIMIC-IV (v3.1) database, we developed and compared five supervised machine learning models to predict a composite outcome of 30-day mortality, prolonged mechanical ventilation (>7 days), or discharge to a skilled nursing or rehabilitation facility (outcome prevalence 56.1%). Predictors were drawn exclusively from routinely available admission-time data, including nocturnal chartevent frequency, nighttime RASS scores, the Hospital Frailty Risk Score (HFRS), comorbidity and severity indices, and standard demographic variables. **Results**. The three gradient boosting models achieved statistically equivalent discrimination (ROC AUC ≈ 0.82–0.83) with strong calibration. SHAP analysis of the best-performing model (CatBoost) identified age, HFRS, nocturnal chartevent count, and nighttime RASS as the most influential predictors, confirming that frailty and sleep fragmentation contribute independently to risk. **Conclusions**. These findings show that EHR-derived frailty signals and nocturnal monitoring intensity, available without additional assessment burden, provide clinically actionable risk stratification at ICU admission and a viable foundation for embedded decision-support tools.

## 1. Introduction

Sleep disorders constitute one of the most pervasive yet systematically overlooked clinical entities encountered during intensive care unit (ICU) admissions. A systematic review and meta-analysis of critically ill patients further demonstrated that sleep disturbance affects 66% of intensive care unit admissions and persists in approximately one-quarter of patients more than a year after discharge, underscoring the durability of these disturbances beyond the acute episode [1]. Despite this burden, sleep complaints are infrequently elicited and even more rarely documented: as Meissner and colleagues showed in a now-classic Veterans Affairs study, fewer than one in five sleep complaints raised by hospitalized patients are recorded in the medical chart, leaving most cases neither investigated nor managed [2]. The clinical consequences of this underdiagnosis are not trivial. Disrupted sleep in hospitalized patients has been causally implicated in the onset and severity of delirium, prolonged length of stay, increased ventilator days, immune dysregulation, cardiometabolic destabilization, and impaired post-discharge functional recovery [3,4]. Reviews of inpatient sleep emphasize a “vicious cycle” in which environmental and iatrogenic disruptors interact with intrinsic illness to produce a clinically meaningful syndrome that worsens patient-reported outcomes and inflates resource use [5,6]. Sleep, therefore, occupies a paradoxical position in modern hospital medicine: it is at once a fundamental physiological process, a frequent comorbidity, and an under-captured determinant of outcomes that merits proactive identification at the point of admission.

A growing body of geriatric and sleep-medicine research has converged on frailty as a key biological substrate that links sleep disturbance to adverse hospital trajectories. Frailty, conceptualized either as a clinical phenotype of reduced physiological reserve [7] or as the cumulative deficit-accumulation index of Rockwood and Mitnitski [8], captures the heterogeneity of biological aging in a way that chronological age alone cannot. Multiple systematic reviews and meta-analyses have established that the relationship between frailty and sleep is bidirectional and graded across multiple sleep dimensions. Pourmotabbed et al. demonstrated that both short (<6 h) and long (>8 h) sleep durations, daytime drowsiness, sleep-disordered breathing, and prolonged sleep latency each independently elevate the odds of frailty [9]. Disease-specific axes complement this general picture: heart failure (ICD-10 I50.x) carries a sleep-disordered-breathing prevalence of 50–80% with reciprocal effects on cardiac remodeling [10]; chronic obstructive pulmonary disease (J44.x) is associated with insomnia in over half of patients and shares autonomic signatures detectable through heart rate variability [11,12]; obesity (E66.x) and type 2 diabetes (E11.x) form an “intertwined trio” with obstructive sleep apnea, with up to 86% of obese patients with diabetes affected [13]; and depression and anxiety (F32.x, F41.x) maintain a robustly bidirectional relationship with insomnia, doubling the odds of incident depression in longitudinal cohorts [14,15]. Frailty, therefore, is not merely a confounder but a clinically interpretable summary of the multisystem vulnerability that gives rise to disordered sleep in hospitalized patients.

Despite this rich biological rationale, methodological gaps remain in translating these insights into actionable inpatient screening tools. The Hospital Frailty Risk Score (HFRS), developed by Gilbert et al. from 109 ICD-10 diagnostic codes, has been validated against the Fried phenotype and Rockwood frailty index and demonstrates fair-to-moderate concordance while uniquely leveraging routinely collected administrative data to flag at-risk inpatients with no additional assessment burden [16]. Subsequent work has refined HFRS construction by combining current and prior admission data and confirmed its predictive validity for length of stay, mortality, and readmission across health systems [17]. The Elixhauser comorbidity index, with ICD-10 mappings standardized by Quan et al., similarly provides a granular comorbidity profile that consistently outperforms alternatives in predicting in-hospital mortality and readmission [18,19]. Administrative-data risk scores such as these are now ubiquitous in hospital epidemiology, but they have rarely been deployed as predictors of sleep-disorder diagnosis. Most existing machine learning (ML) models for sleep disorders rely on polysomnographic data, sleep questionnaires (Epworth Sleepiness Scale, Pittsburgh Sleep Quality Index, Insomnia Severity Index), or wearable signals such as actigraphy and ECG-derived heart rate variability [20,21,22]. While these approaches achieve high discrimination—XGBoost-based models for obstructive sleep apnea reach AUROCs of 0.83–0.87, and hybrid XGBoost-BiLSTM and LightGBM ensembles report comparable performance [23,24,25]—their reliance on inputs that are not routinely available at admission limits scalability for the population of hospitalized patients in whom case findings are most needed. EHR-driven approaches, such as the temporal validation study by Holler et al., have demonstrated that statewide health-information-exchange data can support insomnia prediction with extreme gradient boosting [26], yet to date, no published model has explicitly combined an ICD-coded frailty index, the Elixhauser score, prior healthcare-utilization patterns, vitals, basic laboratory data, and demographics to predict composite adverse outcomes at the moment of ICU admission.

The present study addresses this gap by developing and evaluating a suite of supervised ML models to predict a composite adverse outcome—encompassing 30-day mortality, prolonged mechanical ventilation (>7 days), and discharge to a skilled nursing or rehabilitation facility—using inputs that are reliably available at or shortly after ICU admission. Specifically, we integrate administrative frailty indices (the HFRS and the Elixhauser comorbidity index) and chronological age—itself a strong frailty proxy [8,27]—with objective, routinely captured metrics of nocturnal sleep fragmentation, including the absolute count of nocturnal charted events and nighttime Richmond Agitation-Sedation Scale (RASS) scores. We further incorporate the SOFA score, admission type, and standard demographic variables. We benchmark gradient boosting algorithms—including XGBoost [28] and LightGBM—against random forest and decision tree baselines using nested cross-validation, calibration analysis, and SHAP-based interpretability. Our central hypothesis is that frailty, combined with the intensity of nocturnal monitoring and sedation levels, provides a parsimonious yet biologically grounded representation of multisystem vulnerability and that capturing the nonlinear interactions among these variables will yield clinically actionable predictions of adverse ICU trajectories. By demonstrating that admission-time frailty signals and nocturnal fragmentation metrics can flag high-risk patients, this work aims to lay the methodological foundation for embedded EHR decision-support tools that guide targeted clinical interventions, such as care clustering and sedation optimization, to ultimately improve recovery trajectories for critically ill patients.

## 2. Materials and Methods

### 2.1. Data and Cohort

**Figure 1:**
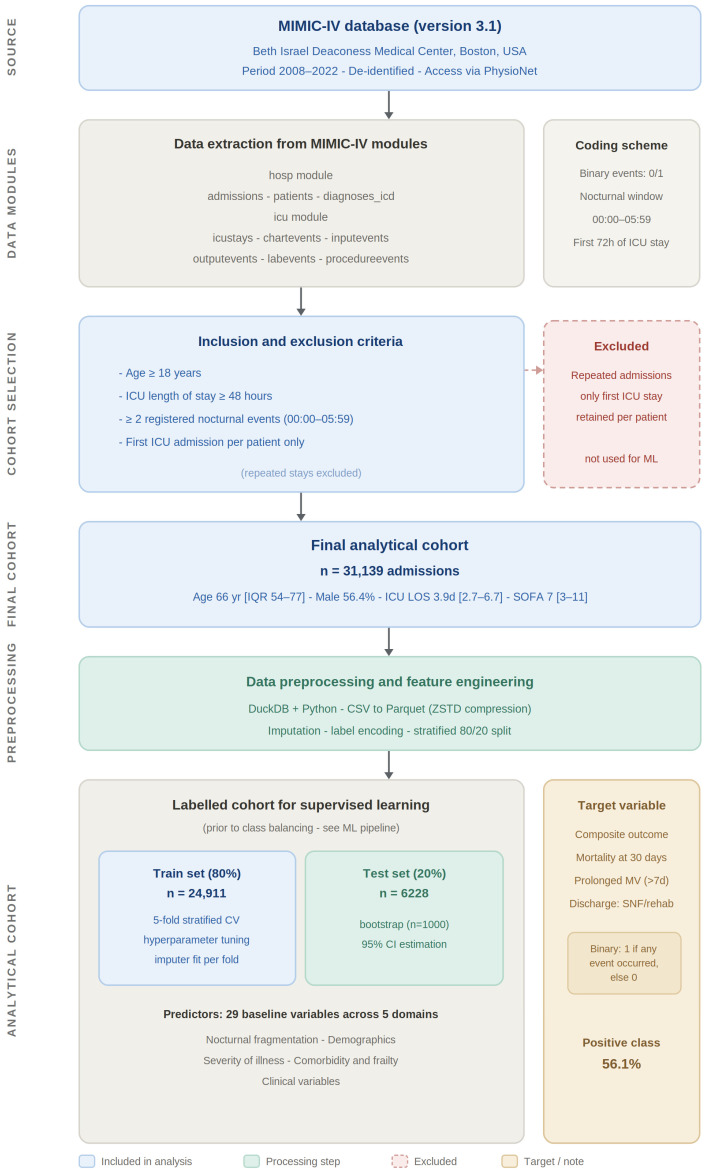
Overview of the FRAGMENT pipeline: data extraction, cohort selection, preprocessing, and supervised learning architecture for ICU adverse outcome prediction using MIMIC-IV.

The overall study design and data processing pipeline are illustrated in Figure 1. The Medical Information Mart for Intensive Care IV (MIMIC-IV, version 3.1) was used as the data source—a publicly available de-identified intensive care database containing clinical records of patients at the Beth Israel Deaconess Medical Center (Boston, MA, USA) for the period from 2008 to 2022. Access to the data was obtained in accordance with the established requirements of PhysioNet. The use of publicly available de-identified data does not require approval from a local ethics committee.

Raw data were extracted from the hosp and icu module tables of the MIMIC-IV database, including admissions, patients, icustays, chartevents, inputevents, outputevents, labevents, procedureevents, and diagnoses_icd, as shown in the data extraction stage of Figure 1. A cohort was formed from the original database following the inclusion and exclusion criteria depicted in the cohort selection block. Inclusion criteria were: age ≥ 18 years, ICU length of stay ≥48 h, and the presence of at least two registered nocturnal events (00:00–05:59) during the first 72 h of ICU stay. The exclusion criterion was the presence of repeated ICU admissions—only the first admission was analyzed for each patient, which allowed for avoiding the violation of the independence of observations. After applying the selection criteria, the final analytical cohort totaled n = 31,139 admissions.

Data preprocessing and feature engineering were performed using Python 3.4.16 and DuckDB 1.5.4, following the pipeline shown in Figure 1. The utilization of DuckDB and the conversion of raw CSV files to Parquet format (with ZSTD compression) were implemented strictly as computational optimizations to efficiently handle the large volume of MIMIC-IV data, rather than as a methodological innovation. This technical approach significantly accelerated memory-intensive data extraction and subsequent analytical queries. The preprocessing stage included imputation, label encoding, and a stratified 80/20 split into training and test sets. The training set (80%, n = 24,911) was used for 5-fold stratified cross-validation and hyperparameter tuning, while the test set (20%, n = 6228) was reserved for bootstrap estimation and final model evaluation.

Baseline characteristics of the cohort are presented in Table 1. The median age was 66 years [IQR 54–77], and the proportion of men was 56.4%. The median ICU length of stay was 3.9 days [IQR 2.7–6.7]. The median SOFA score at admission was 7 [IQR 3–11], reflecting a predominantly moderate-to-severe patient population. The weighted Elixhauser index demonstrated a pronounced right-hand skew (median 0 [IQR 0–14], mean 7.5 ± 10.5), reflecting the heterogeneity of the comorbid burden in the study cohort. The target variable for supervised learning was a composite outcome (mortality at 30 days, prolonged mechanical ventilation >7 days, or discharge to SNF/rehab), which occurred in 56.1% of the cohort (n = 17,469), as indicated in the analytical cohort section of Figure 1. The composite outcome was operationalized as a binary variable equal to 1 if any of the three component events occurred, and 0 otherwise. Component definitions and exact MIMIC-IV extraction logic were as follows: (1) 30-day mortality was defined as death (dod field in the patients table) occurring within 30 days of ICU admission time (admittime); (2) prolonged mechanical ventilation was defined as cumulative invasive ventilation duration exceeding 7 days, computed by summing the duration between starttime and endtime across all procedureevents records with itemid 225792 (Invasive Ventilation) for each ICU stay; (3) discharge to a skilled nursing or rehabilitation facility was identified from the discharge_location field in the admissions table, with values ‘SKILLED NURSING FACILITY’, ‘REHAB’, and ‘CHRONIC/LONG TERM ACUTE CARE’ mapped to a positive outcome. The Hospital Frailty Risk Score was derived from ICD-10 diagnosis codes using the weighted code list of Gilbert et al. (2018), matched against the diagnoses_icd table by ICD-10 code prefix [16].

### 2.2. Feature Engineering

The final feature set included 29 variables grouped into five thematic blocks: nocturnal sleep fragmentation, demographics and anthropometry, severity of illness, comorbidity and frailty, and clinical variables. A comprehensive list of all 29 features, including their clinical definitions, MIMIC-IV source tables, data types, and pre-imputation missingness rates, is provided in Appendix B.

All nocturnal exposure variables were calculated within the first 72 h of the ICU stay and were limited to the nocturnal time window of 00:00–05:59. This approach ensured temporal separation between the exposure and outcomes, excluding reverse causality. The selection of the 00:00–05:59 time window was guided by three converging rationales. First, from a circadian biology perspective, this interval corresponds to the physiologically most vulnerable sleep period: under normal entrainment, melatonin secretion peaks near 03:00 and endogenous cortisol concentrations reach their nadir between approximately 00:00 and 04:00, defining the core biological rest window during which slow-wave sleep (N3) and REM sleep are predominantly concentrated [29]. Disruption of this window is therefore expected to produce the greatest derangement in restorative sleep architecture. Second, from a clinical operations perspective, the 00:00–05:59 interval avoids the confounding effects of evening handover activities (typically 22:00–24:00), during which chartevent density transiently increases due to shift-change documentation rather than genuine patient care, thereby improving the specificity of chartevent counts as a proxy for nocturnal monitoring intensity. Third, prior ICU sleep-disruption research has validated a broadly equivalent nocturnal window (22:00–06:00) as the standard observation period for nocturnal nursing interactions [30,31,32], and our 00:00–05:59 definition represents a conservative subset of this window that excludes the highest-noise portion of the night. Sensitivity analyses using an extended 22:00–05:59 window yielded qualitatively identical feature importance rankings supporting the robustness of this definitional choice. The primary indicator of fragmentation was the average number of chartevents per night (events_per_night), supplemented by the absolute number of nocturnal events (n_night_events), the frequency of vital signs recording (n_night_vitals), and the number of nocturnal lab draws (n_night_lab_draws). Sedation was assessed using the RASS: mean (rass_mean_night) and minimum (rass_min_night) nocturnal values were calculated, as well as the number of deep sedation episodes (RASS ≤ 3; n_deep_sedation_night). A binary missingness flag was generated for patients without nocturnal RASS records. Among the study cohort, 13.9% of patients lacked nocturnal RASS documentation. This flag was explicitly included as a model feature, as the absence of sedation monitoring may itself reflect illness severity, nursing workload, or a clinical decision that the patient did not require sedation assessment—all of which carry prognostic relevance. Additionally, the number of nocturnal administrations of sedatives, analgesics, and vasopressors (inputevents) was taken into account.

The analysis included age, sex, body mass index (BMI), admission type, and ICU profile (first_careunit). BMI was calculated based on the earliest weight and height measurements in chartevents. Missing height values were filled with the sex-stratified median; missing BMI values were recalculated based on the imputed anthropometric data.

Severity was assessed using the complete six-component SOFA score, calculated for the first 24 h of the ICU stay based on the worst values of each parameter within this window. Components included: respiratory (PaO_2_/FiO_2_ ratio), coagulation (platelets), hepatic (total bilirubin), cardiovascular (mean arterial pressure and vasopressor use), neurological (Glasgow Coma Scale), and renal (creatinine and daily urine output) values. In cases where vasopressor use was recorded without infusion rate data in \mu g/kg/min, the cardiovascular component was scored as 3 points—this simplification was pre-specified in the study protocol. This approach assigns the same cardiovascular SOFA subscore to all patients receiving any vasopressor regardless of agent or dose intensity and may therefore introduce a systematic upward bias in SOFA for patients on low-dose vasopressors whose true cardiovascular component would be 2 rather than 3. The direction of this bias is conservative with respect to severity estimation: affected patients would be assigned a higher SOFA than their actual infusion rates would justify, leading to a marginal overestimation of illness acuity in that subset. The proportion of admissions affected by this imputation was limited to those with documented vasopressor use but absent rate data in the inputevents table, and the pre-specified assignment was applied uniformly to avoid post hoc adjustments. A formal sensitivity analysis comparing model discrimination and SHAP feature importance rankings between the full cohort and a restricted cohort excluding patients with missing vasopressor rate data is acknowledged as an important validation step that was not performed within the scope of the present study and is recommended prior to clinical deployment of the model. Additionally, maximum HR, minimum systolic blood pressure, and minimum SpO_2_ during the first 24 h were included as features.

Comorbid burden was assessed using the weighted Elixhauser index based on ICD-10 codes assigned during the current hospitalization. To avoid circularity, the primary discharge diagnosis (seq_num = 1) was not included in the index calculation. This exclusion is methodologically necessary because the primary discharge diagnosis (seq_num = 1) represents the principal reason for the current hospitalization and is therefore determined by the clinical course rather than being a pre-existing comorbidity. Including it in the Elixhauser index would introduce circularity, as it may directly reflect the very adverse outcome being predicted. Frailty was assessed using the Hospital Frailty Risk Score (HFRS), calculated from ICD-10 codes. The number of previous hospitalizations was used as an additional marker of the patient’s functional reserve. The primary diagnosis category (3-digit ICD-10 code) was included as a categorical feature to account for the nosological structure of the cohort.

Variables with a semantic zero in the absence of a value (nocturnal medications, vasopressors, HFRS, Elixhauser) were filled with zero prior to the dataset split. Categorical gaps in the primary diagnosis were replaced with the label “Unknown.” Residual missing values for continuous variables (vital signs, SOFA components) were addressed via median imputation implemented within a scikit-learn Pipeline. This ensured that the imputer was fit exclusively on the training folds during each cross-validation iteration, with the learned medians subsequently applied to the held-out validation fold. This approach prevents validation fold information from leaking into imputation statistics, eliminating a common source of optimistic bias in reported cross-validation performance. Categorical variables were encoded using LabelEncoding, with the encoder trained on the train set and applied to the test set; unknown categories in the test set were assigned a value equal to the number of known categories (i.e., one beyond the encoded range), placing them outside the trained encoding space to avoid spurious tree splits on a sentinel value. The proportion of unseen categories in the test set was less than 0.5%, confirming that this assignment had negligible influence on model behavior.

### 2.3. ML Models

To address the binary classification task, five machine learning models were trained and compared: Decision Tree, Random Forest, XGBoost, CatBoost, and LightGBM. This selection was driven by the need to cover both interpretable baseline algorithms and modern gradient boosting methods that demonstrate consistently high efficiency on tabular clinical data. Hyperparameter tuning for all models was performed using randomized search (RandomizedSearchCV) with 5-fold stratified cross-validation, the ROC AUC target metric, and a fixed random_state = 42 for reproducibility.

The Decision Tree served as a baseline interpretable model, providing a benchmark to evaluate the performance gains of more complex algorithms. The model builds a hierarchical structure of binary splits in the feature space, selecting at each node the feature and threshold that minimize a specified impurity criterion. The hyperparameter search space included: splitting criterion (gini, entropy), maximum tree depth (None, 10, 20, 30), minimum number of samples required to split a node (2, 5, 10), minimum number of samples in a leaf (1, 2, 4), and the number of features considered at each split (None, sqrt, log2). The search was conducted over 100 iterations.

Random Forest is an ensemble method based on bagging that trains multiple decision trees on bootstrap samples and subsequently averages their predictions. The introduction of a random subset of features at each split reduces the correlation between trees and decreases model variance compared to a single tree. The search space included: the number of trees (50, 100), maximum depth (None, 10, 20), minimum samples for a split (2, 5), minimum samples in a leaf (1, 2), number of features per split (sqrt, log2), and the use of bootstrapping (True, False). The search was conducted over 50 iterations.

XGBoost implements regularized gradient boosting on decision trees with explicit control over model complexity via L1/L2 regularization, a minimum loss reduction parameter (gamma), and a minimum leaf weight (min_child_weight). The algorithm sequentially adds trees, each approximating the residuals of the previous step using a second-order Taylor expansion of the loss function.

The search space included: learning rate (0.01, 0.05, 0.1, 0.2), maximum tree depth (3, 5, 7, 10), number of trees (100, 200, 500), subsample ratio (0.6, 0.8, 1.0), feature fraction (0.6, 0.8, 1.0), min_child_weight (1, 3, 5), and gamma (0, 0.1, 0.2). The search was conducted over 100 iterations.

CatBoost implements an ordered boosting algorithm that eliminates gradient estimate bias by using only those observations for each object that precede it in a random permutation of the training set. A key advantage of CatBoost for this study is its native handling of categorical features without prior encoding, which is particularly relevant given high-cardinality variables such as the primary diagnosis (primary_icd_category) and ICU profile (first_careunit). The search space included: number of iterations (100, 250), learning rate (0.05, 0.1), tree depth (4, 6, 8), L2 regularization coefficient (1, 3, 5), number of split borders (32, 64, 128), and tree growth strategy (SymmetricTree, Depthwise). The search was conducted over 50 iterations.

LightGBM utilizes two key algorithmic solutions that distinguish it from classical gradient boosting: GOSS (Gradient-based One-Side Sampling), which retains objects with large gradients and randomly samples objects with small gradients during tree construction, and EFB (Exclusive Feature Bundling), which bundles exclusive features to reduce dimensionality. Together, these mechanisms provide a significant acceleration in training with minimal loss in quality. Additionally, LightGBM builds trees using a leaf-wise strategy (as opposed to the level-wise strategy in XGBoost), allowing it to achieve lower error with the same number of leaves. The search space included: number of leaves (31, 50, 80), maximum depth (−1, 5, 7, 10), learning rate (0.05, 0.1, 0.2), number of trees (100, 200), minimum number of samples in a leaf (20, 50, 100), subsample ratio (0.6, 0.8), and feature fraction (0.6, 0.8). The search was conducted over 30 iterations.

All experiments are reproducible with a fixed seed (random_state = 42). The implementation was carried out in Python using the scikit-learn, XGBoost, CatBoost, and LightGBM libraries. The final hyperparameter search spaces for all models are presented in Appendix A.

### 2.4. Validation Strategy

To ensure correct assessment of the models’ generalization ability and prevent data leakage, a multi-level validation strategy was applied, including a split into training and test sets, stratified cross-validation, and bootstrap estimation of confidence intervals.

In the first stage, the original cohort was split into training (80%, n = 24,911) and test (20%, n = 6228) sets using stratified random splitting to maintain class proportions in both parts. All imputation, categorical variable encoding, and scaling procedures were performed exclusively on the training set, with the learned transformations subsequently applied to the test set. The test set remained completely isolated throughout the entire process of hyperparameter tuning and model training.

In the second stage, hyperparameter tuning was conducted via 5-fold stratified cross-validation (StratifiedKFold) on the training set. Stratification by the target variable ensured a balanced class distribution in each fold. The optimization target metric was the Area Under the Receiver Operating Characteristic curve (ROC AUC) as the measure of discriminative power most robust to class imbalance. After determining the optimal hyperparameters, each model was retrained on the full training set and finally evaluated on the test set.

In the third stage, a bootstrap method (n = 1000 iterations) was applied to the test set to obtain unbiased metric estimates with confidence intervals. In each iteration, a sample of the same size as the test set was formed with replacement, followed by the calculation of a complete set of performance metrics. Final point estimates and 95% confidence intervals were determined as the mean and the 2.5–97.5 percentiles of the bootstrap statistic distribution. Iterations in which the bootstrap sample contained fewer than two unique classes were excluded from the calculation. Across all models, the number of excluded iterations was zero in all 1000 bootstrap runs, consistent with the 56.1% composite outcome prevalence rendering single-class samples practically impossible.

Additionally, for each final model, 5-fold cross-validation was performed on the full training set, calculating the mean and standard deviation for all six quality metrics. Comparing the cross-validation results and the bootstrap estimates on the test set allowed for the detection of possible overfitting and an assessment of model stability.

### 2.5. Evaluation Metrics

Model quality was evaluated using six metrics covering various aspects of classification performance and clinical applicability.

In all formulas, TP denotes true positives, TN—true negatives, FP—false positives, and FN—false negatives.

*Accuracy* reflects the proportion of correctly classified observations among all objects in the test set. Despite the widespread use of this metric, its interpretation requires caution with imbalanced classes; therefore, it was considered in conjunction with other indicators.

(1) $$Accuracy=\frac{TP+TN}{TP+TN+FP+FN}\;$$

*Precision* (positive predictive value) determines the proportion of true positive predictions among all observations classified by the model as positive. A high precision value indicates a low rate of false positives, which is important for avoiding inappropriate interventions.

(2) $$Precision=\frac{TP}{TP+FP}$$

*Recall* (sensitivity) determines the proportion of true positive observations correctly identified by the model among all actual positive cases. In a clinical context, high recall means a minimal rate of missed patients with an adverse outcome.

(3) $$Recall=\frac{TP}{TP+FN}$$

The *F*1-*score* is the harmonic mean of precision and recall, providing a balanced evaluation of the model when both indicators are equally important. This metric was one of the primary measures used for comparing models alongside ROC AUC.

(4) $$F1=2\times \frac{Precision\times Recall}{Precision+Recall}$$

*ROC AUC* (area under the receiver operating characteristic curve) represents the probability that the model will assign a higher predictive score to a randomly chosen positive observation than to a randomly chosen negative one. *ROC AUC* is independent of the selected classification threshold and robust to moderate class imbalance; thus, it was used as the primary metric for hyperparameter tuning and final model comparison. The optimal classification threshold was determined using Youden’s J statistic (the maximum difference between sensitivity and specificity on the ROC curve)

(5) $$ROC\;AUC=\int_{0}^{1}TPR\left(t\right)d(FPR(t))=p({\hat{p}}_{+}>{\hat{p}}_{-})$$

where ${\hat{p}}_{+}$ and ${\hat{p}}_{-}$ are the predicted probabilities for a randomly selected positive and negative observation, respectively.

(6) $$t*=argmax\;[TPR\;\left(t\right)-FPR\;\left(t\right)]$$

*PR AUC* (area under the precision–recall curve) is an additional metric particularly informative for imbalanced classes. Unlike *ROC AUC*, *PR AUC* is sensitive to changes in model performance regarding the positive class and allows for a more detailed assessment of ranking quality in the clinically significant region of high recall.

(7) $$PR\;AUC=\sum_{k=1}^{n}P_{k}*(R_{k}-R_{k-1})$$

where $P_{k}$ and $R_{k}$ denote precision and recall at the kk k-th classification threshold.

For all six metrics, point estimates on the test set were accompanied by 95% confidence intervals obtained using the bootstrap method (n = 1000 iterations), as well as mean values and standard deviations from the results of 5-fold cross-validation on the training set. The combined use of both approaches ensured an assessment of both the generalization ability of the model and the stability of its predictions.

### 2.6. Interpretability

Model interpretability was ensured at two levels: global, characterizing the overall importance of features for the model as a whole, and local, explaining the contribution of each feature to an individual prediction.

At the global level, built-in mechanisms of gradient boosting models were used to assess the relative importance of features. The mean absolute SHAP value across the entire test set was used as a measure of importance, ensuring a consistent and theoretically grounded comparison of feature contributions regardless of the specifics of a particular algorithm. The results were visualized as a horizontal bar chart displaying the top 15 most significant features.

At the local level, the SHAP (SHapley Additive exPlanations) method was applied, based on Shapley’s cooperative game theory. The SHAP value of each feature for a specific observation is defined as its weighted average contribution to the model’s prediction relative to the base value (the average prediction across the training set), calculated across all possible feature coalitions. This approach satisfies the axioms of local accuracy, missingness (absence of dummy features), and symmetry, ensuring a theoretically sound and consistent decomposition of the prediction.

For the final CatBoost model, SHAP values were calculated on the test set using TreeExplainer—an efficient implementation for the exact calculation of SHAP for tree-based models with a complexity of O(TLD2), where T is the number of trees, L is the number of leaves, and D is the tree depth. The results were presented in two visualization formats: a summary dot plot, displaying the distribution of SHAP values for each feature across all test set observations with color-coding of the actual feature values, and a summary bar plot, showing the mean absolute SHAP value as an aggregated measure of global importance. Both plots were limited to the top 15 features to ensure readability. To quantify the interaction between nocturnal event count and sedation level, SHAP dependence plots were generated for n_night_events and rass_mean_night with cross-coloring by the complementary feature. The Pearson correlation between n_night_events and its SHAP values was additionally computed separately for patients in the upper RASS quartile (light sedation or agitation, RASS ≥ Q3) and the lower quartile (deep sedation, RASS ≤ Q1).

The combined use of built-in feature importance and SHAP analysis allowed for verifying the consistency of results between the two methods and provided a clinically interpretable explanation of model predictions in the context of the investigated problem of nocturnal sleep fragmentation in the ICU.

## 3. Results

In the framework of this study, five machine learning models were trained and compared to predict a composite adverse outcome in ICU patients. The results of the cross-validation and bootstrap estimation on the test set are presented in Table 2. All gradient boosting models (XGBoost, CatBoost, LightGBM) significantly outperformed the baseline Decision Tree model across all metrics. Random Forest occupied an intermediate position, demonstrating a ROC AUC of 0.817 during cross-validation.

The best ROC AUC results during cross-validation were achieved by the LightGBM model (0.8296 ± 0.0068); however, its advantage over CatBoost (0.8292 ± 0.0072) and XGBoost (0.8274 ± 0.0059) was not statistically significant. This is confirmed by the complete overlap of the 95% confidence intervals obtained via the bootstrap method: LightGBM 0.8250 [0.8145–0.8354], CatBoost 0.8237 [0.8136–0.8344], and XGBoost 0.8227 [0.8119–0.8335]. A similar pattern was observed for PR AUC: LightGBM 0.854 ± 0.0064, CatBoost 0.8530 ± 0.0074, and XGBoost 0.8518 ± 0.0058. Given the practical equivalence of the three gradient boosting models and considering CatBoost’s advantages in native categorical feature handling, the CatBoost model was selected for the subsequent interpretability analysis.

**Figure 2:**
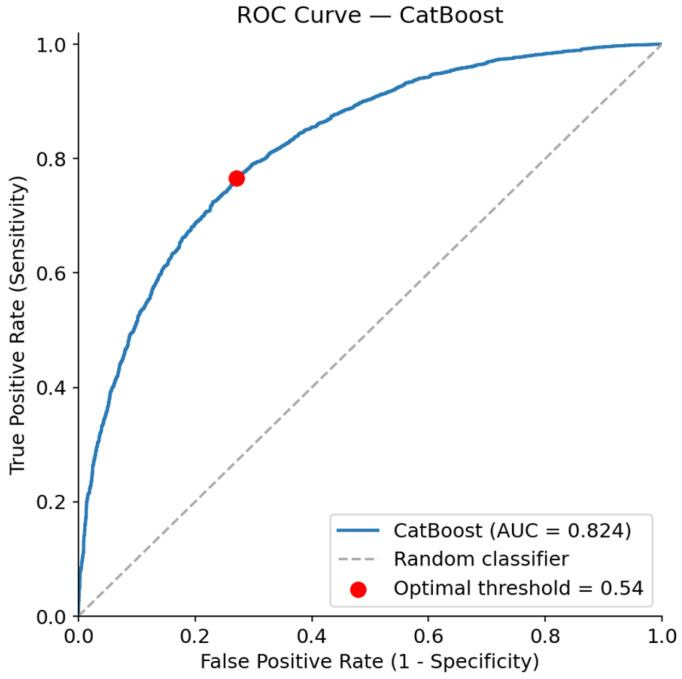
ROC curve and area under the curve (AUC) for the CatBoost predictive model on the test set.

The ROC curve of the CatBoost model on the test set is shown in Figure 2. The area under the ROC curve was 0.824, indicating good discriminative power of the model. The optimal classification threshold, determined by Youden’s J statistic, was 0.54, corresponding to a point with a sensitivity of 0.796 and a specificity of 0.687.

**Figure 3:**
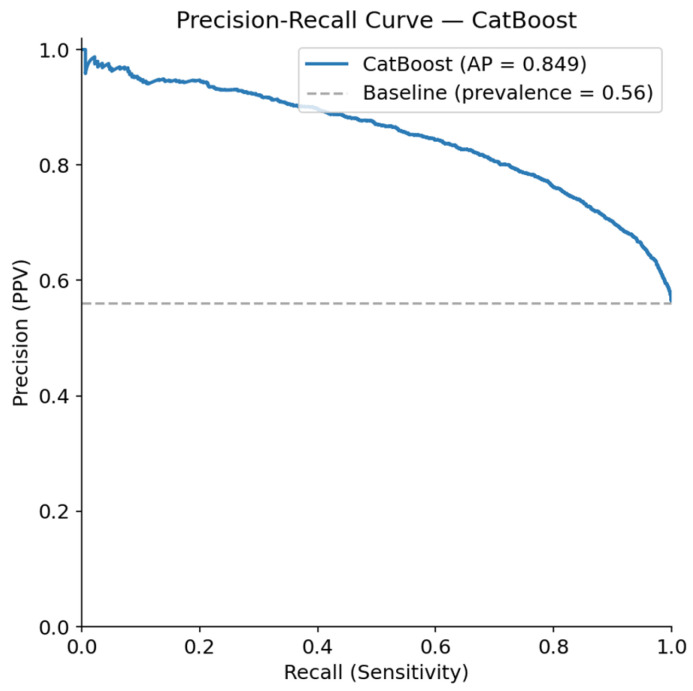
Precision–Recall curve for the CatBoost model on the test set, with an Average Precision (AP) of 0.849.

The Precision–Recall curve (Figure 3) was characterized by an area under the curve of AP = 0.849, which significantly exceeds the baseline level of a random classifier (prevalence = 0.56). The high initial precision at low recall values and the gradual decline of the curve indicate robust ranking of positive observations by the model.

**Figure 4:**
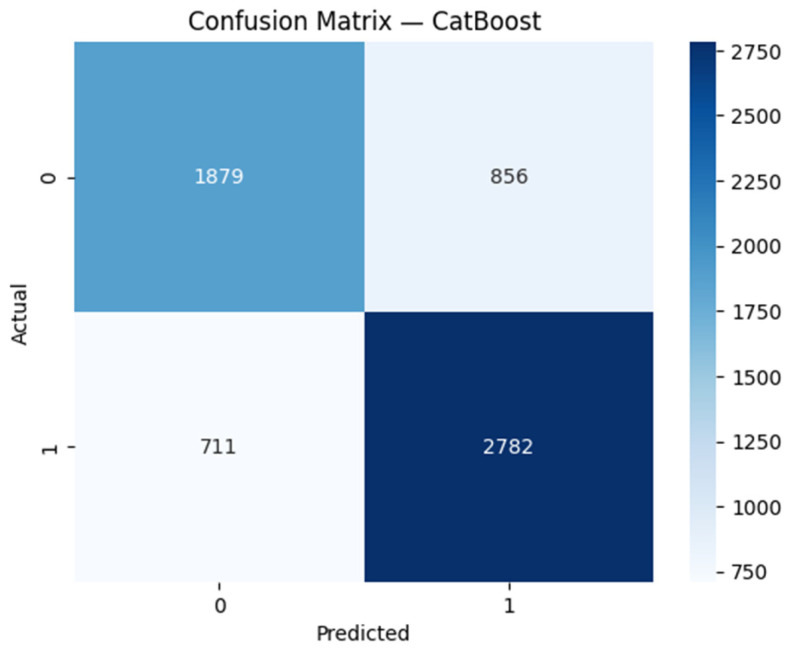
Confusion matrix for the CatBoost model on the test set.

The confusion matrix at a threshold of 0.54 (Figure 4) demonstrates the following distribution of predictions on the test set (n = 6228): 2782 true positives, 1879 true negatives, 711 false negatives, and 856 false positives. The final clinical performance characteristics of the model are: sensitivity 0.796, specificity 0.687, positive predictive value (PPV) 0.765, and negative predictive value (NPV) 0.725.

**Figure 5:**
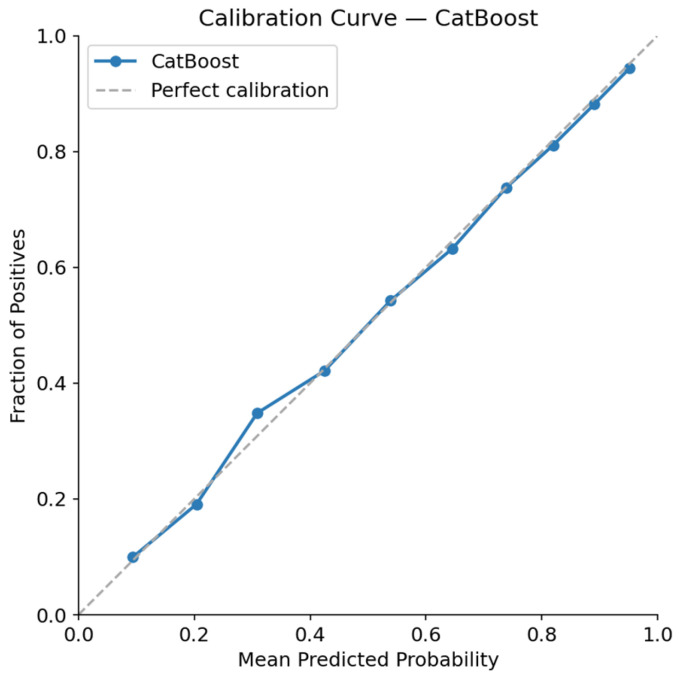
Calibration curve comparing predicted probabilities with actual outcomes.

The calibration curve of the CatBoost model (Figure 5) demonstrates close agreement between predicted probabilities and observed event rates across the full prediction range. To quantify calibration formally, three metrics were computed on the held-out test set: the Brier score was 0.169 (reference no-skill score: 0.250), indicating substantially better-than-chance probabilistic accuracy. Logistic calibration regression of the binary outcome on the logit of predicted probabilities yielded a calibration intercept of 0.008 (ideal: 0) and a calibration slope of 0.959 (ideal: 1.0), confirming minimal systematic bias and only slight overestimation of predicted risk, consistent with the minor deviation observed in the 0.20–0.30 probability range. Overall, calibration can be characterized as good, supporting the use of predicted probabilities for individualized risk communication.

**Figure 6:**
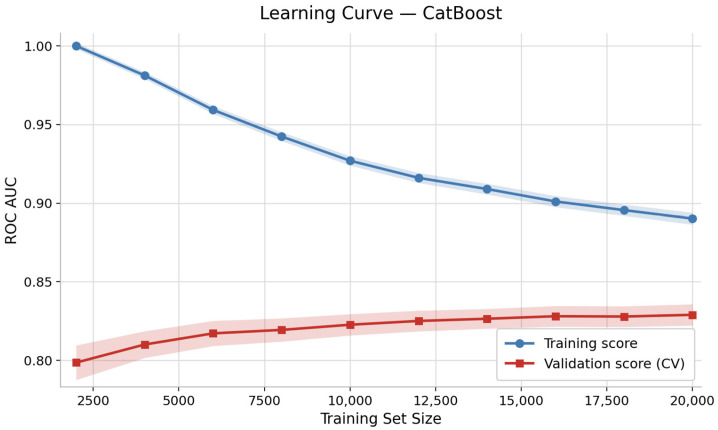
Learning curve showing training and validation ROC AUC scores.

The learning curve (Figure 6) illustrates the ROC AUC dynamics as a function of the training sample size. The training score monotonically decreases from 1.000 at n = 2500 to 0.889 at n = 20,000, which indicates a gradual reduction in overfitting as the data volume increases. The validation score (CV) demonstrates steady growth from 0.800 to 0.830 and reaches a plateau at a sample size of approximately 10,000–12,500 observations, after which the performance gain slows down. The presence of a stable gap between the training and validation scores (\sim0.06 with the full dataset) reflects the ability, typical of ensemble methods, to memorize training patterns while maintaining high generalization ability on the holdout set. The stabilization of the validation score indicates that the current sample size is sufficient for robust model training. The learning curve was generated using 5-fold stratified cross-validation at each of six subset sizes (n = 2491; 4982; 9964; 14,946; 19,928; 24,911), with the Pipeline-embedded imputer refit independently within each fold at each subset size to prevent leakage.

**Figure 7:**
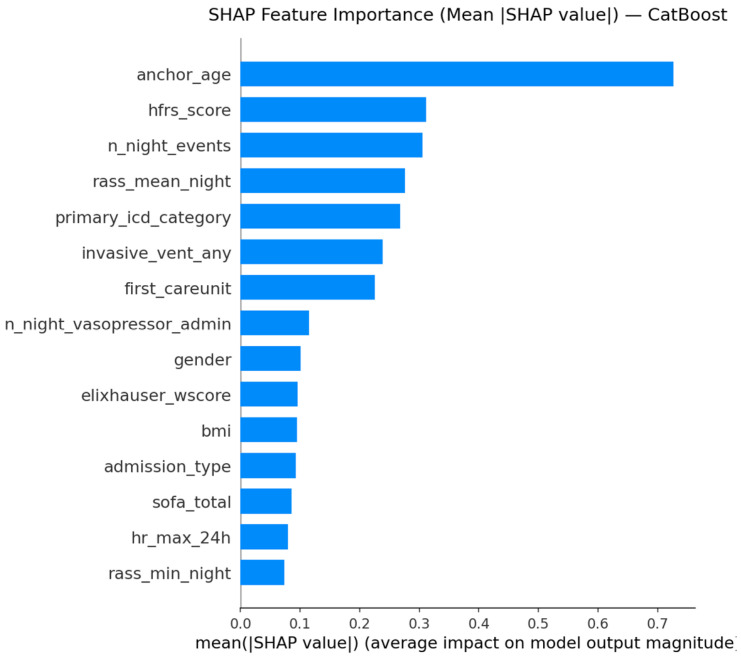
Feature importance ranking based on mean absolute SHAP values.

**Figure 8:**
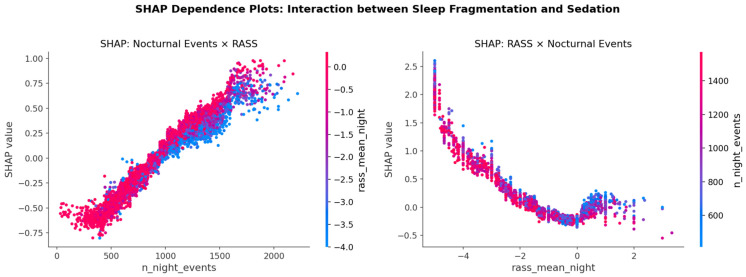
SHAP dependence plots illustrating the interaction between nocturnal event count (n_night_events) and mean nocturnal RASS score (rass_mean_night). Left panel: SHAP values for n_night_events colored by rass_mean_night. Right panel: SHAP values for rass_mean_night colored by n_night_events.

Feature importance analysis using the SHAP method (Figure 7 and Figure 8) revealed a stable hierarchy of predictors for the adverse outcome. The greatest contribution to the model’s predictions was made by patient age (anchor_age; mean |SHAP| ≈ 0.73), significantly outpacing all other features. The SHAP summary plot demonstrates that advanced age (red dots) is consistently associated with positive SHAP values—increasing the predicted probability of an adverse outcome—whereas younger age (blue dots) exerts the opposite protective effect.

The second most significant feature was the Hospital Frailty Risk Score (hfrs_score; mean |SHAP| ≈ 0.31), confirming the role of frailty as an independent predictor of adverse outcomes regardless of age. Third place was held by the absolute number of nocturnal events in chartevents (n_night_events; mean |SHAP| ≈ 0.30), a key variable of nocturnal fragmentation. This is a central result of the study, supporting the hypothesis that the intensity of nocturnal monitoring is linked to adverse outcomes.

**Figure 9:**
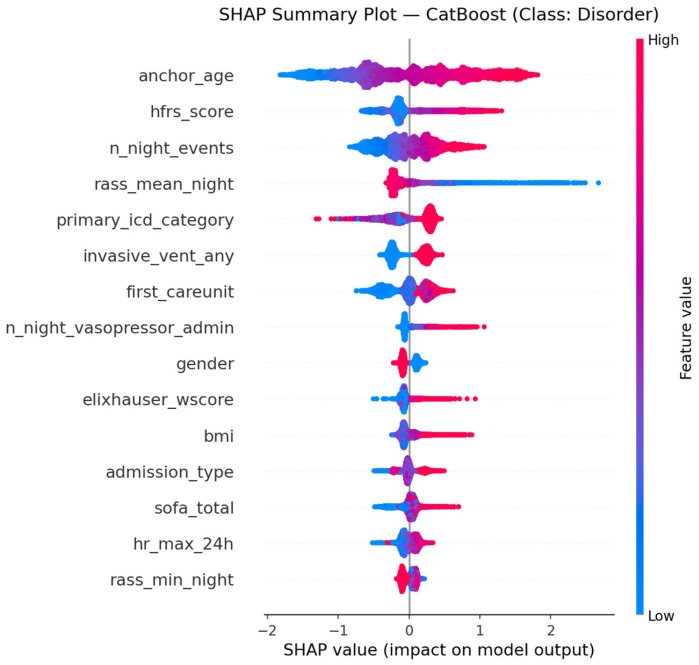
SHAP summary plot showing the impact of feature values on model output.

The mean nocturnal RASS value (rass_mean_night; mean |SHAP| ≈ 0.28) ranked among the top four features. The SHAP summary plot reveals the nonlinear nature of this relationship: high RASS values (less negative, indicating lighter sedation or agitation; red dots) are associated with pronounced positive SHAP values reaching +2.5, while low RASS values (deep sedation; blue dots) are generally associated with a neutral or moderately negative contribution. Quantification of the interaction between nocturnal event count and sedation level revealed that the Pearson correlation between n_night_events and its SHAP values was r = 0.978 in patients with light sedation or agitation (RASS ≥ Q3) and r = 0.945 in deeply sedated patients (RASS ≤ Q1; Δr = 0.033), as shown in Figure 9. The minimal difference indicates that the predictive contribution of nocturnal event count is largely independent of sedation depth.

The primary diagnosis category (primary_icd_category; mean |SHAP| ≈ 0.27) and ICU type (first_careunit; mean |SHAP| ≈ 0.23) reflected the nosological and organizational heterogeneity of the cohort. The presence of invasive ventilation (invasive_vent_any; mean |SHAP| ≈ 0.24) ranked sixth, highlighting the significance of the severity of respiratory failure. The lower portion of the top 15 included the weighted Elixhauser index (elixhauser_wscore), BMI, admission type (admission_type), SOFA score (sofa_total), and maximum heart rate during the first 24 h (hr_max_24h). Notably, sofa_total, despite its clinical relevance, occupied a relatively low position (mean |SHAP| ≈ 0.08), which may be explained by the partial overlap of information with other severity-of-illness features already included in the model.

## 4. Discussion

In this study, five machine learning models were compared for predicting adverse outcomes in ICU patients. Three gradient boosting algorithms—XGBoost, CatBoost, and LightGBM—demonstrated significantly superior performance: cross-validated ROC AUC values ranged from 0.827 to 0.830, and bootstrap estimation on the test set yielded partially overlapping 95% confidence intervals. The high performance of gradient boosting algorithms aligns with recent systematic reviews examining ICU clinical prediction models: a 2025 systematic review analyzing artificial intelligence and ML models for predicting in-hospital mortality in the ICU identified XGBoost, Random Forest, and logistic regression as the most frequently used algorithms [33]. In a German ICU cohort, a dynamic LightGBM model achieved an AUROC of 0.859 during external validation on MIMIC-IV data [34]; while this exceeds the performance of our static model based solely on admission-time data, the difference is attributable to the use of time-series features updated every 24 h in that study.

Given the absence of statistically significant differences among the three boosting models, CatBoost was selected for interpretability analysis due to its ability to handle categorical variables without prior encoding. SHAP analysis provided clinically plausible, hierarchically structured explanations for the model’s predictions. Patient age emerged as the most influential predictor (mean |SHAP| ≈ 0.73): advanced age increased the probability of adverse outcomes. This finding aligns with the cumulative deficit model of biological aging conceptualized within the Rockwood–Mitnitski framework. Notably, the Hospital Frailty Risk Score (HFRS) ranked second (mean |SHAP| ≈ 0.31), functioning as an independent predictor beyond age. This result is corroborated by validation data for HFRS in the ICU context: in a Canadian multicenter cohort of 22,178 patients, those in the high-frailty-risk group exhibited significantly higher rates of mortality and ICU readmission [35]. External validation of HFRS among elderly patients receiving mechanical ventilation demonstrated associations between medium and high-risk groups and poorer outcomes, including prolonged hospitalization and 30-day readmission [36]. A study using MIMIC data in ICU patients with atrial fibrillation further confirmed substantially higher in-hospital mortality (18.8% vs. 7.6%) and 30-day mortality (24.5% vs. 12.3%) in the frail group [37].

The absolute count of nighttime charted events ranked third (mean |SHAP| ≈ 0.30)—this represents the core indicator of sleep fragmentation, forming the conceptual foundation of this study. This finding is consistent with evidence examining the association between sleep disruption and adverse outcomes in ICU patients. A review investigating sleep and delirium in critically ill ICU patients highlighted that sleep fragmentation—i.e., sleep distributed across the 24 h cycle—is associated with delirium, prolonged hospitalization, and increased morbidity, with up to 50% of sleep occurring during daytime hours [4]. In a 2024–2025 prospective accelerometry study, mortality reached 45% in groups with prolonged or new-onset delirium, significantly higher than the 13.3% observed in the non-delirious group [38]. A systematic review encompassing 19 studies on ICU patient sleep confirmed that ICU patients frequently experience medical and nursing interventions, with vital sign monitoring being the most common, and that care clustering protocols demonstrate effectiveness [31]. A survey on quality of care in the ICU found that patients experienced an average of 19.47 ± 4.20 nighttime interruptions and that awakenings for diagnostic/therapeutic procedures significantly prolonged sleep latency [39].

The mean nighttime RASS value (mean |SHAP| ≈ 0.28) further reinforced the model’s clinical coherence. SHAP plots revealed a nonlinear relationship: light sedation or an agitated state was associated with higher predicted risk, whereas deep sedation showed neutral or slightly protective contributions. This pattern aligns with evidence examining the dual risks of sedation regarding outcomes. In a prospective multicenter cohort using MIMIC-IV data, deep sedation was associated with higher mortality and significantly fewer ventilator-free and ICU-free days, though an agitated state also emerged as an independent mortality factor [40]. Additionally, a study using MIMIC-IV data determined that low RASS variability during the first 72 h of ICU stay was associated with a 1.57-fold increase in 28-day mortality risk [41].

### 4.1. Practical Implications

From the perspective of clinical applicability and predictive operational characteristics, at the optimal threshold of 0.54 determined by Youden’s J statistic, sensitivity was 0.796, specificity 0.687, PPV 0.765, and NPV 0.725. The selection of this threshold, and the sensitivity-specificity trade-off it implies, warrants explicit clinical interpretation. In the ICU context, the primary priority for a risk-stratification tool is to minimise missed high-risk patients—that is, to keep false negatives low—because failure to identify a deteriorating patient carries greater consequences than the administrative burden of a false alarm that triggers unnecessary monitoring review. A sensitivity of 0.796 means that approximately four in five patients who will genuinely experience an adverse outcome are correctly flagged at admission, enabling early initiation of targeted interventions such as care clustering, sedation optimisation, and frailty-oriented multidisciplinary care. The corresponding specificity of 0.687 implies that approximately 31% of truly low-risk patients are incorrectly classified as high-risk; at the PPV of 0.765, however, roughly three in four patients flagged by the model do indeed experience an adverse outcome, limiting the proportion of clinically unnecessary alerts. This trade-off is broadly consistent with the asymmetric cost structure of ICU triage: the consequences of under-treating a high-risk patient substantially outweigh the cost of additional vigilance for a patient who recovers uneventfully. Importantly, the threshold of 0.54 was derived from the overall test set using Youden’s J statistic to balance sensitivity and specificity at the population level; clinical deployment could permit institution-specific threshold adjustment—for example, lowering the threshold to increase sensitivity further in high-acuity units where the cost of missed deterioration is highest, or raising it in lower-acuity settings to reduce alert burden. Such recalibration would require prospective validation in the target institutional context. Given that approximately two-thirds of patients classified as high-risk indeed experienced adverse outcomes, these results support the early initiation of multidisciplinary, frailty-oriented care. The calibration curve demonstrated close agreement between predicted probabilities and observed outcomes, confirming the reliability of predictions for clinical decision-making purposes. The HFRS emerging as the second most influential predictor—independent of age and acute illness severity—supports the systematic incorporation of frailty screening into ICU admission workflows using routinely available administrative data, without requiring additional assessment burden. The strong predictive signal from nocturnal chartevent frequency provides quantitative, EHR-derived support for care-clustering and sleep-preservation protocols at the point of admission: patients flagged as high-risk by the model can be prospectively enrolled in targeted nocturnal care bundles designed to reduce unnecessary monitoring interactions and protect restorative sleep. Because all model inputs are available at or shortly after ICU admission without requiring polysomnography, wearable sensors, or dedicated sleep-medicine infrastructure, the approach is scalable across resource-diverse hospital settings and could be embedded as an automated alert within existing EHR platforms with minimal additional infrastructure.

A critical epistemological consideration warrants explicit discussion beyond the acknowledgement of limitations alone. The absolute count of nocturnal chartevents (n_night_events), despite ranking as the third most influential predictor in SHAP analysis (mean |SHAP| ≈ 0.30), is not a direct physiological characteristic of the patient but rather a behavioural output of nursing practice that is systematically modulated by illness severity—a pattern broadly analogous to confounding by indication in pharmacoepidemiology. In the ICU, monitoring intensity is a clinician-determined response to perceived acuity: more deteriorated patients receive more frequent vital sign checks, laboratory draws, and medication titrations, all of which generate chartevents, creating a partially circular pathway: higher acuity, more nocturnal monitoring events, higher predicted risk. Three converging considerations, however, support interpreting n_night_events as a clinically informative signal rather than a mere severity surrogate. First, the model already incorporates multiple direct severity measures—SOFA score, RASS, invasive ventilation status, and the Elixhauser index—so the incremental SHAP contribution of n_night_events (mean |SHAP| ≈ 0.30, comparable to HFRS at 0.31 and substantially exceeding SOFA at 0.08) reflects information not captured by these conventional acuity indices. This is consistent with the broader EHR informatics literature: Agniel and colleagues demonstrated across 272 laboratory test types that the presence and frequency of clinical ordering events carry independent prognostic value above and beyond the test results themselves because clinician decisions to order tests encode the latent severity signals that structured severity scores do not fully capture [42]. Second, the phenomenon of monitoring frequency as an independent predictor is well established under the term “informative observation”: the rate at which data are collected is itself informative about the underlying clinical state, and this informativeness is not equivalent to circularity when the predictive goal is risk stratification rather than causal attribution [43]. Third, the clinical and biological interpretation of n_night_events extends beyond illness acuity: for a given severity level, patients who receive more nocturnal interventions experience greater sleep fragmentation, with documented downstream consequences including delirium, prolonged mechanical ventilation, and impaired immune recovery—pathways that independently mediate adverse outcomes irrespective of the initial severity that prompted the monitoring [4,31]. The study design is therefore best understood not as claiming that nocturnal chartevent counts cause adverse outcomes, but rather that they constitute an accessible EHR-derived composite signal integrating both illness acuity and iatrogenic sleep disruption and that this composite signal provides clinically actionable risk stratification. Causal disentanglement of these two components would require instrumental variable methods or a prospective design with randomised monitoring protocols—an important direction for future work.

### 4.2. Limitations

This study has several limitations that must be acknowledged. The cohort was derived exclusively from the MIMIC-IV database (Beth Israel Deaconess Medical Center, Boston, MA, USA), a tertiary academic medical centre with a predominantly urban patient population, specific nursing staffing ratios, and a particular EHR documentation culture—all of which may systematically differ from community hospitals, rural ICUs, non-US healthcare systems, and populations with different demographic or comorbidity profiles. This single-centre origin is not a minor caveat but a substantive constraint on the present model’s readiness for clinical deployment: a model trained on BIDMC chartevent patterns, HFRS distributions, and outcome rates cannot be assumed to perform equivalently in settings where monitoring intensity norms, frailty prevalence, or composite outcome rates differ. This concern is reinforced by the broader ML literature, in which only 14.7% of health-related machine learning studies validate models on independent external data [44] and, by evidence, that MIMIC-IV-trained models can show meaningful performance degradation on external cohorts even within the same country [45]. External validation on geographically and institutionally diverse datasets—including eICU-CRD, AmsterdamUMCdb, and ideally at least one non-English-speaking healthcare system—is therefore a prerequisite for clinical deployment rather than an optional future step and should be explicitly addressed before the model is considered for integration into any institutional decision-support workflow. Second, nocturnal chartevent counts represent indirect, EHR-derived proxies of sleep disruption rather than direct objective assessments of sleep architecture, and it is not possible on the basis of the present data alone to fully disentangle whether monitoring frequency reflects clinical severity, iatrogenic sleep disruption, or both; residual confounding by indication cannot be excluded despite the inclusion of multiple direct severity covariates in the model. Third, the composite outcome combines three clinically distinct endpoints that differ substantially in both prevalence and clinical significance: 30-day mortality (16.0%), prolonged mechanical ventilation > 7 days (10.0%), and discharge to a skilled nursing or rehabilitation facility (39.6%). This prevalence asymmetry means that the composite is dominated numerically by the SNF/rehab discharge component, which carries different clinical stakes and a different predictor profile than mortality or prolonged ventilation. Frailty indices such as HFRS may preferentially predict functional decline and post-acute care placement, whereas acute severity markers such as SOFA and invasive ventilation status more plausibly drive mortality and ventilator-days endpoints. By pooling these outcomes, the composite model may learn a weighted average of their predictor structures and mask differential associations that are clinically meaningful: a predictor genuinely informative for mortality risk but not for functional discharge—or vice versa—will have its SHAP contribution attenuated in the composite framework. Decomposing the outcome into its three components via separate models or a multi-task learning architecture that shares a common representation while fitting distinct output heads per endpoint would allow each predictor’s contribution to be evaluated at the endpoint-specific level. This is prioritised as an important direction for future work. Fourth, the cardiovascular component of the SOFA score was assigned a fixed value of 3 for all patients with documented vasopressor use but missing infusion rate data, representing a pre-specified simplification that may introduce conservative upward bias in SOFA estimation for a subset of the cohort. A sensitivity analysis excluding these patients was not conducted and is recommended as a validation step before clinical deployment. Fifth, all model inputs were drawn from the first 72 h of ICU admission, and the model therefore does not capture the dynamic evolution of fragmentation and sedation over longer stays. Sixth, the classification threshold of 0.54 was selected using Youden’s J statistic on the test set, and optimal threshold calibration for deployment in a specific ICU would require prospective validation in that institutional context.

### 4.3. Future Research

Several important directions exist for future research. Most critically, and as a prerequisite for any clinical deployment, multi-institutional external validation must be performed using datasets from academic centres and general hospitals both within and outside the United States—including eICU-CRD, AmsterdamUMCdb, and cohorts from non-English-speaking healthcare systems—with pre-specified acceptable performance thresholds (e.g., AUC ≥ 0.80, calibration slope 0.90–1.10) defined prior to validation rather than post hoc. Decomposing the composite outcome into its individual components would enable the model to distinguish between mortality and functional discharge trajectories. From a technical standpoint, temporal deep learning architectures—such as transformer-based sequential models—could better capture the dynamic interplay among fragmentation, sedation, and physiological deterioration over the full ICU stay. Methodologically, instrumental variable approaches or prospective randomised monitoring protocols are needed to causally disentangle the independent contributions of illness acuity and iatrogenic sleep disruption to the nocturnal chartevent signal. Finally, the most important translational objective is to embed model outputs into clinical decision support systems and conduct randomised controlled trials testing whether model-guided nursing interventions—including care clustering, sleep bundles, and optimised sedation protocols—reduce the incidence of adverse outcomes in flagged high-risk patients. This study presents an ML-based framework integrating frailty and sleep fragmentation within a unified analytical system for ICU clinical practice and establishes the methodological and empirical foundation for that translational programme.

## 5. Conclusions

This study demonstrates that machine learning models integrating frailty indices and nocturnal sleep fragmentation metrics derived from routinely collected electronic health record data can reliably predict composite adverse outcomes—encompassing 30-day mortality, prolonged mechanical ventilation, and discharge to post-acute care—in a large heterogeneous ICU cohort. Among the five algorithms evaluated, gradient boosting methods (XGBoost, CatBoost, and LightGBM) achieved statistically equivalent and clinically meaningful discrimination, with bootstrap-estimated ROC AUC values of approximately 0.82–0.83, substantially outperforming the Decision Tree baseline. SHAP-based interpretability analysis of the best-calibrated model identified patient age, the Hospital Frailty Risk Score, absolute nocturnal chartevent frequency, and mean nighttime RASS score as the four dominant predictors, underscoring the complementary contributions of biological aging, cumulative deficit burden, and iatrogenic sleep disruption to adverse ICU trajectories. Importantly, all model inputs are available at or shortly after admission without requiring additional clinical assessments, polysomnography, or dedicated sleep-medicine infrastructure, which makes the approach readily scalable across diverse hospital settings.

These results carry direct clinical implications. The HFRS emerging as the second most influential predictor—independent of age and acute illness severity—supports the systematic incorporation of frailty screening into ICU admission workflows. The strong predictive signal from nocturnal chartevent frequency supports further investigation of care-clustering and sleep-preservation protocols as candidate nursing interventions, pending prospective validation of their causal impact on outcome risk. The model’s well-characterized calibration further enables its use as a probability estimator rather than a simple classifier, facilitating individualized risk communication and targeted resource allocation.

Several limitations must be acknowledged. The cohort is restricted to a single academic center in the United States, and generalizability to community hospitals, non-English-speaking healthcare systems, or populations with different demographic composition remains to be established. Nocturnal chartevent counts represent indirect proxies of sleep disruption that cannot substitute for objective sleep architecture assessment. The composite outcome, while clinically meaningful, aggregates mechanistically distinct endpoints whose individual predictions warrant separate investigation. Future research should prioritize multi-institutional external validation using datasets such as eICU-CRD or AmsterdamUMCdb, decomposition of the composite outcome into its constituent components, and incorporation of longitudinal time-series features to capture dynamic physiological deterioration. Ultimately, prospective randomized trials testing whether model-guided nursing interventions—including sleep bundles, optimized sedation protocols, and care clustering—translate the predictive signal identified here into measurable reductions in adverse events will be essential to establish the clinical utility of this approach.

## Figures and Tables

**Table 1:** Baseline characteristics of the study cohort (n = 31,139).

Characteristic	Value
Demographics	
Age, median [IQR], years	66 [54–77]
Male sex, n (%)	17,550 (56.4%)
ICU stay	
Length of stay, median [IQR], days	3.9 [2.7–6.7]
Severity and frailty scores	
SOFA score (first 24 h), median [IQR]	7 [3–11]
Elixhauser weighted score, median [IQR]	0 [0–14]
Hospital Frailty Risk Score, median [IQR]	1.5 [0–10.4]
Outcomes	
Composite outcome, n (%)	17,469 (56.1%)
30-day mortality	4982 (16.0%)
Prolonged mechanical ventilation (>7 days)	3114 (10.0%)
Discharge to SNF/rehab	12,331 (39.6%)

**Table 2:** Comparative analysis of classification metrics across different machine learning algorithms.

Model	Evaluation Method	Accuracy	F1	Precision	Recall	ROC AUC	PR AUC
Decision Tree	Cross-validation (mean ± SD)	0.706 ± 0.003	0.740 ± 0.002	0.735 ± 0.007	0.745 ± 0.010	0.761 ± 0.004	0.775 ± 0.006
	Bootstrap test set (95% CI)	0.701 [0.689–0.712]	0.728 [0.715–0.740]	0.743 [0.727–0.757]	0.713 [0.698–0.727]	0.757 [0.745–0.770]	0.769 [0.751–0.784]
Random Forest	Cross-validation (mean ± SD)	0.745 ± 0.005	0.781 ± 0.004	0.754 ± 0.005	0.810 ± 0.004	0.817 ± 0.005	0.841 ± 0.004
	Bootstrap test set (95% CI)	0.736 [0.725–0.747]	0.773 [0.761–0.784]	0.747 [0.733–0.760]	0.800 [0.786–0.813]	0.811 [0.800–0.822]	0.836 [0.822–0.850]
XGBoost	Cross-validation (mean ± SD)	0.753 ± 0.004	0.784 ± 0.005	0.768 ± 0.003	0.802 ± 0.008	0.827 ± 0.006	0.852 ± 0.006
	Bootstrap test set (95% CI)	0.746 [0.735–0.757]	0.778 [0.767–0.789]	0.762 [0.748–0.776]	0.796 [0.782–0.808]	0.823 [0.812–0.834]	0.848 [0.835–0.861]
CatBoost	Cross-validation (mean ± SD)	0.756 ± 0.006	0.788 ± 0.006	0.769 ± 0.004	0.808 ± 0.010	0.829 ± 0.007	0.853 ± 0.007
	Bootstrap test set (95% CI)	0.748 [0.737–0.760]	0.780 [0.770–0.791]	0.765 [0.750–0.778]	0.797 [0.783–0.810]	0.824 [0.814–0.834]	0.849 [0.837–0.861]
LightGBM	Cross-validation (mean ± SD)	0.754 ± 0.007	0.786 ± 0.007	0.767 ± 0.006	0.805 ± 0.009	0.830 ± 0.007	0.854 ± 0.006
	Bootstrap test set (95% CI)	0.747 [0.736–0.757]	0.780 [0.769–0.791]	0.760 [0.746–0.773]	0.801 [0.788–0.814]	0.825 [0.815–0.835]	0.849 [0.835–0.861]

## Data Availability

The data used in this study are sourced from the MIMIC-IV database, which is publicly available on PhysioNet (https://doi.org/10.13026/6mm1-ek67). Access to the database is restricted to credentialed users who have completed the CITI ‘Data or Specimens Only Research’ training and signed the Data Use Agreement. The complete analytical code, including SQL extraction scripts, preprocessing pipelines, and model training/evaluation algorithms, is available from the corresponding author upon reasonable request.

## Abbreviations

The following abbreviations are used in this manuscript:

AP	Average Precision
BiLSTM	Bidirectional Long Short-Term Memory
BMI	Body Mass Index
CRP	C-Reactive Protein
CV	Cross-Validation
EFB	Exclusive Feature Bundling
EHR	Electronic Health Record
eICU-CRD	eICU Collaborative Research Database
FN	False Negative
FP	False Positive
FPR	False Positive Rate
GCS	Glasgow Coma Scale
GOSS	Gradient-based One-Side Sampling
HFRS	Hospital Frailty Risk Score
HR	Heart Rate
HRV	Heart Rate Variability
ICU	Intensive Care Unit
IL-6	Interleukin-6
IQR	Interquartile Range
MIMIC-IV	Medical Information Mart for Intensive Care IV
ML	Machine Learning
NPV	Negative Predictive Value
PaO_2_/FiO_2_	Ratio of Arterial Partial Pressure of Oxygen to Fractional Inspired Oxygen
PPV	Positive Predictive Value
PR AUC	Area Under the Precision–Recall Curve
RASS	Richmond Agitation-Sedation Scale
ROC AUC	Area Under the Receiver Operating Characteristic Curve
SHAP	SHapley Additive exPlanations
SNF	Skilled Nursing Facility
SOFA	Sequential Organ Failure Assessment
SpO_2_	Peripheral Oxygen Saturation
TNF-α	Tumor Necrosis Factor-alpha
TN	True Negative
TP	True Positive
TPR	True Positive Rate

## Appendix A

**Table A1:** Hyperparameter search spaces.

Model	Hyperparameters
Decision Tree	max_depth: {None, 10, 20, 30}; min_samples_split: {2, 5, 10}; min_samples_leaf: {1, 2, 4}; criterion: {gini, entropy}; max_features: {None, sqrt, log2}; n_iter = 100
Random Forest	n_estimators: {50, 100}; max_depth: {None, 10, 20}; min_samples_split: {2, 5}; min_samples_leaf: {1, 2}; max_features: {sqrt, log2}; bootstrap: {True, False}; n_iter = 50
XGBoost	learning_rate: {0.01, 0.05, 0.1, 0.2}; max_depth: {3, 5, 7, 10}; n_estimators: {100, 200, 500}; subsample: {0.6, 0.8, 1.0}; colsample_bytree: {0.6, 0.8, 1.0}; min_child_weight: {1, 3, 5}; gamma: {0, 0.1, 0.2}; n_iter = 100
CatBoost	iterations: {100, 250}; learning_rate: {0.05, 0.1}; depth: {4, 6, 8}; l2_leaf_reg: {1, 3, 5}; border_count: {32, 64, 128}; grow_policy: {SymmetricTree, Depthwise}; n_iter = 50
LightGBM	num_leaves: {31, 50, 80}; max_depth: {−1, 5, 7, 10}; learning_rate: {0.05, 0.1, 0.2}; n_estimators: {100, 200}; min_child_samples: {20, 50, 100}; subsample: {0.6, 0.8}; colsample_bytree: {0.6, 0.8}; n_iter = 30

## Appendix B

**Table A2:** Feature Specification, Definitions, and Data Sources from the MIMIC-IV Database.

Thematic Block	Feature Name	Definition/Clinical Meaning	MIMIC-IV	Data Type
Demographics & Anthropometry	anchor_age	Patient age at the time of ICU admission	patients	Continuous
	gender	Patient sex (Male/Female)	patients	Categorical
	bmi	Body Mass Index, calculated from earliest weight and height	chartevents	Continuous
	weight_kg	Patient weight in kilograms	chartevents	Continuous
	height_cm	Patient height in centimeters	chartevents	Continuous
Admission Profile	admission_type	Type of hospital admission (e.g., Urgent, Emergency)	admissions	Categorical
	first_careunit	First intensive care unit type the patient was admitted to	icustays	Categorical
Comorbidity & Frailty	hfrs_score	Hospital Frailty Risk Score	diagnoses_icd	Continuous
	elixhauser_wscore	Weighted Elixhauser comorbidity index	diagnoses_icd	Discrete
	prior_hospitalizations	Count of previous hospital admissions	admissions	Discrete
	primary_icd_category	Primary ICD-10 diagnosis category	diagnoses_icd	Categorical
Severity of Illness (First 24 h)	sofa_total	Sequential Organ Failure Assessment (SOFA) score	Multiple	Discrete
	hr_max_24h	Maximum heart rate within the first 24 h	chartevents	Continuous
	sbp_min_24h	Minimum systolic blood pressure within the first 24 h	chartevents	Continuous
	spo2_min_24h	Minimum peripheral oxygen saturation within the first 24 h	chartevents	Continuous
	pf_ratio	PaO_2_/FiO_2_ ratio (respiratory function)	chartevents/ labevents	Continuous
	gcs_min	Minimum Glasgow Coma Scale score	chartevents	Continuous
Intensive Care Interventions	invasive_vent_any	Presence of invasive mechanical ventilation (binary)	procedureevents	Binary
	vasopressor_any	Administration of any vasopressor (binary)	inputevents	Binary
	vasopressor_total_amount	Total dose of administered vasopressors	inputevents	Continuous
Nocturnal Sleep Fragmentation	events_per_night	Average number of charted events per night	chartevents	Continuous
(Measured between 00:00–05:59)	n_night_events	Absolute count of nocturnal charted events	chartevents	Discrete
	n_night_vitals	Count of nocturnal vital sign recordings	chartevents	Discrete
	n_night_lab_draws	Count of nocturnal laboratory blood draws	labevents	Discrete
	n_night_sedative_admin	Count of nocturnal sedative administrations	inputevents	Discrete
	n_night_analgesic_admin	Count of nocturnal analgesic administrations	inputevents	Discrete
	n_night_vasopressor_admin	Count of nocturnal vasopressor administrations	inputevents	Discrete
Sedation Status	rass_mean_night	Mean Richmond Agitation-Sedation Scale score at night	chartevents	Continuous
	rass_min_night	Minimum RASS score at night	chartevents	Continuous
	n_deep_sedation_night	Number of nights spent in deep sedation (RASS ≤ −3)	chartevents	Discrete
	rass_missing_flag	Binary indicator for missing nocturnal RASS records	Derived	Binary
